# Medical insurance, livelihood capital and public health in China

**DOI:** 10.1186/s12962-024-00554-z

**Published:** 2024-05-28

**Authors:** Wang Sheng, Liao Fuchong

**Affiliations:** 1 School of Business Administration, Northeastern University, Shenyang City, Liaoning Province China; 2 School of Public Administration, Central South University, 220th Shaoshan Road, Tianxin District, Changsha City, Hunan Province China

**Keywords:** Population health, The Healthy China Strategy, Family livelihood, Medical coverage

## Abstract

**Background:**

Medical insurance stands as a pivotal component within the overarching framework of public service systems. The intricate interplay between the extent of healthcare coverage and the overall well-being of the populace remains a pivotal research question within the academic sphere.

**Methods:**

Drawing from the comprehensive dataset of the Chinese Household Livelihood Survey, this article employs a rigorous data model to delve into the profound implications of medical coverage on population health.

**Results:**

The descriptive analysis revealed that areas with broader medical coverage tend to exhibit higher levels of overall population health. This initial observation provided a foundation for further quantitative exploration using multiple regression analysis. The regression analysis demonstrated a statistically significant positive relationship between medical coverage and population health. This finding is particularly noteworthy as it suggests that expanding access to healthcare services has tangible benefits for improving the overall health of a population.

**Conclusion:**

From the lens of familial sustenance, this article delves into the intricate health implications of medical coverage, thereby introducing a novel theoretical lens to the evolving discourse surrounding medical insurance healthcare systems and their impact on public health. This approach aims to enrich the current understanding of this complex relationship and contribute to the scholarly dialogue.

## Introduction

The Healthy China Strategy is a significant public service program that the Chinese government is vigorously promoting, exerting widespread influence. Among its components, the establishment of a medical coverage serves as a crucial part of the Healthy China Strategy. The construction of China's medical coverage is constantly advancing. Many inspiring research conclusions have been proposed regarding the policy effects of healthcare coverage on population health.

Specifically, in the context of China's poverty alleviation efforts, medical coverage has played a pivotal role. Consequently, the approach to poverty reduction in basic healthcare has shifted its emphasis from resource concentration towards supporting registered impoverished households [1]. Specifically, this shift involves achieving tiered burden reduction through the foundational medical security system, ultimately aiming to mitigate residents' medical expenditures. By continually refining and enhancing the fundamental medical security system that encompasses both urban and rural residents as well as employees, China has gradually forged an effective interface between the achievements of healthcare-oriented poverty reduction and rural revitalization policies [2, 3]. Furthermore, this framework, which encompasses care for the elderly, medical support for the infirm, and poverty assistance, constitutes a critical policy initiative in forestalling large-scale poverty relapse. Simultaneously, it aligns with the ongoing pursuit of enhancing and expanding the social security system. This article delves into the impact of foundational medical coverage on population health.

Unfortunately, there are two shortcomings in existing literature: firstly, there is a lack of research on reducing population health through basic healthcare coverage. The main issue of poverty governance after 2020 is population health [4]. The paramount policy concern lies in identifying strategies to solidify and broaden the successes achieved in poverty alleviation, particularly through social security measures encompassing basic healthcare coverage. While much of the current research surrounding healthcare coverage's impact on poverty reduction has been conducted within the framework of absolute poverty thresholds and poverty reduction initiatives, there remains a notable absence of focus on vital policy matters such as managing citizens' health and defining the role healthcare coverage can assume in the aftermath of China's victory against poverty [5]. Hence, further reinforcement in these areas is urgently required.

Secondly, to a considerable extent, the current research landscape has overlooked the profound influence of family structural factors on population health. The choice to enroll in healthcare coverage remains predominantly an individual-level decision, and numerous studies have primarily concentrated on the individual-level poverty alleviation effects of healthcare coverage [6], thereby neglecting the pivotal role of family structural factors. While certain pertinent studies have endeavored to incorporate structural variables such as family size, family dependency ratio, and household head characteristics in their exploration of the poverty reduction impacts of basic healthcare coverage, a cohesive analytical framework is still lacking, and the theoretical underpinnings of their model construction necessitate further consolidation and reinforcement.

Therefore, within the framework of sustainable livelihoods, this article explores the impact of healthcare coverage on population health, which has two main significance: at the level of theoretical enhancement, by incorporating household structural factors such as livelihood asset and livelihood strategies into the discussion of the poverty reduction effect of basic healthcare coverage [7], it enhances the understanding of the poverty reduction effect of healthcare coverage and expands the theoretical boundary of population health governance. At the level of policy significance, this article focuses on the impact of healthcare coverage on population health and proposes corresponding policy recommendations for the governance of population health after winning the battle against poverty. It has certain policy significance for improving the efficiency of population health governance and further achieving common prosperity.

The remaining part of the article are organized as follows: Section II presents a comprehensive literature review and the establishment of the theoretical framework. Section III delineates the research methodology, encompassing the application of econometric techniques, the dataset employed, and the variables pertinent to the study. Section IV provides an empirical examination of the subject matter, while Section V concludes the discourse with the principal findings and a subsequent discussion.

## Literature review and theoretical framework

### Poverty alleviation and medical coverage in China

The impact of basic healthcare coverage on the health status of insured individuals is a well-established finding in existing research. With regard to the underlying mechanisms of this effect, the extant literature primarily centers on three principal domains:

First, from the perspective of medical service accessibility, hospitals, medical institutions, services, and healthcare coverage jointly form the foundational support for comprehensive medical and health public services. The healthcare system reform in Fujian Province has significantly enhanced the availability of public medical services and achieved notable reform outcomes through the establishment of a governance framework that fosters a synergistic relationship among hospitals, medications, and healthcare coverage, dubbed the "three-pronged medical linkage." Indeed, the healthcare coverage fund serves as a pivotal instrument in the allocation of resources for medical public services. By managing this fund, it facilitates the effective implementation of the hierarchical diagnosis and treatment system, thereby enhancing the quality and standards of grassroots-level public services. The hierarchical diagnosis and treatment system designates specific medical institutions to be responsible for diagnosis and treatment based on the severity of illnesses. This system aims to minimize the time between illness onset and diagnosis, thereby improving accessibility to fundamental medical and health services. Typically, community medical points within township health centers handle most routine illnesses affecting urban and rural residents. However, in cases where primary medical institutions are unable to provide adequate treatment, patients are referred to urban hospitals or provincial-level medical institutions [8]. However, due to various reasons, the current hierarchical diagnosis and treatment in China is seriously "upside down", which is embodied in that the tertiary hospitals absorb most of the patients and medical resources, and the primary medical institutions are difficult to play a role. Due to the high level of medical care, large-scale tertiary hospitals have a strong siphon effect on patients and resources. healthcare coverage can promote the implementation of hierarchical diagnosis and treatment system through the expense reimbursement mechanism, so as to improve the level of health security and cut off the vicious circle from disease to poverty. In fact, China's medical coverage has strict medical treatment procedures and healthcare coverage catalogue, which can play a significant role in guiding residents' medical treatment behavior and help the implementation of hierarchical diagnosis and treatment system. healthcare coverage tamps the availability of public services for primary health care and can play a good health effect.

Second, From the perspective of rational medical practices, it is noteworthy that inhabitants of rural China tend to adopt non-scientific approaches when seeking medical attention. The prevalent trend of "postponing minor ailments and ignoring critical conditions" has become a primary healthcare strategy among rural communities. The frequent occurrence of untreated illnesses poses significant health risks, often leading to the aggravation of minor health issues [9]. This trend is largely attributed to the pervasive concern among rural inhabitants regarding the financial implications of medical expenses. In fact, the long-standing dearth of comprehensive healthcare coverage in rural regions, coupled with the limited economic resources of rural households, has heightened their sensitivity towards the costs of medical care. The reimbursement system for basic healthcare coverage has, to a degree, elevated the eagerness of insured individuals to seek treatment, mitigating concerns associated with hospital visits for diagnostic and therapeutic purposes, thereby preventing potential health deterioration due to delayed medical attention. The existence of such a medical coverage instills a certain psychological assurance among impoverished households, ensuring they receive a proportionate reimbursement and compensation for medical expenses. Based on research conducted on China's New Rural Cooperative Medical System, it is evident that insured farmers exhibit a significantly higher likelihood of hospital visits for examination and treatment when faced with physical discomfort or disease risks, compared to uninsured farmers Crucially, a crucial component of the health poverty alleviation initiative lies in attaining universal healthcare coverage for impoverished households through viable investment strategies. The central budget has allocated special transfer payments to contiguous impoverished regions, facilitating inclusive access to fundamental medical services for these households. Objectively, this poverty alleviation project has notably improved the health status and human capital accumulation of impoverished families by guiding them towards scientific medical practices. Consequently, the medical coverage, to a certain extent, alleviates rural residents' concerns regarding medical expenses, effectively promoting scientific medical behaviors and timely treatment in the event of illness. By encouraging insured individuals to adopt scientific medical practices, the medical coverage exerts significant health benefits.

Third, healthcare coverage disrupts the pernicious cycle of illness and poverty, effectively arresting the descent into poverty triggered by sickness and the subsequent relapse into impoverishment. While examining the impact of health social security on impoverished households occasioned by illness, it becomes evident that within the theoretical framework of sustainable livelihoods, health status serves as a pivotal component of livelihood outcomes. These outcomes are the culmination of households' production and operational endeavors, executed through tailored livelihood strategies, which are themselves shaped by the stock of livelihood assets. Within this theoretical framework, livelihood assets, too, constitute a stock that significantly influences both livelihood strategies and their ultimate outcomes.

In China, the foundational medical coverage system encompasses multiple variations, tailored to address the multifaceted demands of its citizenry. Chief among these are the urban employee basic healthcare coverage and the comprehensive healthcare scheme for urban and rural residents. These two schemes, while interconnected in their overall objectives, exhibit distinct characteristics in terms of their scope of coverage and administrative frameworks. Urban employee basic medical coverage is primarily targeted at employed urban residents, covering workers in urban areas and their dependents. It is a contributory system, where both employees and their employers contribute towards the insurance fund. This system provides comprehensive coverage for inpatient care, outpatient services, and chronic diseases, with relatively high reimbursement rates.

On the other hand, urban and rural basic medical coverage covers both urban and rural residents who are not employed or not covered by urban employee basic medical coverage. This insurance is typically funded through government subsidies and individual contributions. While the coverage may not be as extensive as urban employee basic medical coverage, it still provides vital marketable support for basic medical expenses, especially for those in rural and underserved areas. The relationship between these two types of insurance is complementary. Together, they form a comprehensive medical safety net that aims to provide basic medical protection to all Chinese citizens. While urban employee basic medical coverage focuses on the employed urban population, urban and rural basic medical coverage extends coverage to the broader rural and urban uninsured populations. This arrangement ensures that no matter where one resides or their employment status, they have access to some form of basic healthcare coverage.

**Fig. 1:**
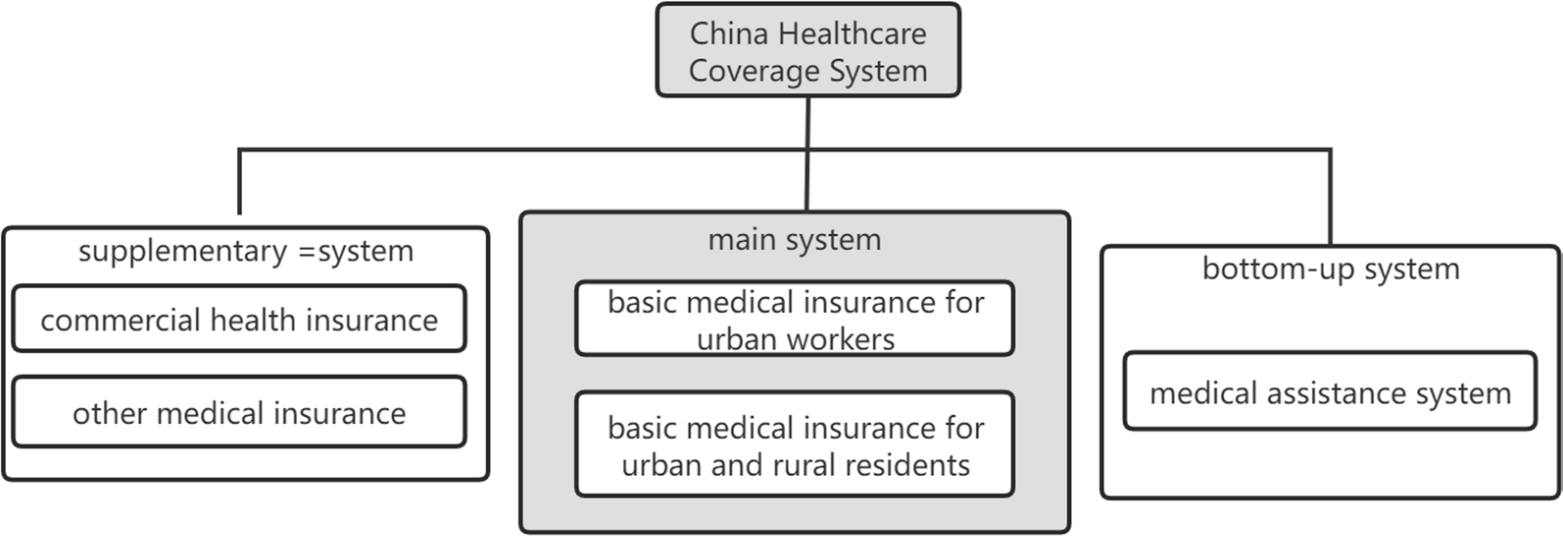
Medical coverage in China

Furthermore, the Chinese government has undertaken initiatives to facilitate the integration of diverse medical insurance schemes, with the overarching goal of optimizing system efficiency and enhancing its overall functionality. This integration process entails the harmonization of policies, standards, and administrative protocols across varying insurance categories, thereby fostering a more consolidated and equitable medical coverage system （Figure 1）.

In conclusion, the basic medical coverage in China is diverse and comprehensive, encompassing multiple types of insurance that are interrelated and complementary. This system, while constantly evolving and improving, plays a crucial role in ensuring that all Chinese citizens have access to basic medical care and marketable protection against the marketable burden of healthcare expenses.

### Theoretical framework

By treating public services as a livelihood asset, the theoretical application scope of the sustainable livelihood framework has been expanded from the perspective of macro institutional and micro household livelihood interaction, which has had a significant impact.

On the basis of existing sustainable livelihood theories, this research suggests that public services are an important public asset for livelihood [10, 11]. There are significant differences and certain similarities between public asset and private asset. On the one hand, the difference is mainly reflected in the fact that public asset mainly refers to public services. Public asset is a decision made by external factors and families, mainly determined by the public sector, while families cannot influence the supply level and distribution mode of public asset. The specific forms of private asset such as material asset, marketable asset, community resource, human asset, and ecological asset are directly related to the livelihood activities of families.

On the other hand, from the vantage point of governmental public policy formulation, the primary factors influencing the policy process encompass public sector attributes, lobbying efforts from the private sector, legal frameworks, regulatory systems, and cultural value norms. Once public decisions are formulated, they exert a direct influence on the level of supply and distribution methods for public service assets. Additionally, these decisions indirectly impact private livelihood assets. The sustainable livelihood framework, when viewed through the lens of public services, rests on two key premises: firstly, the household operates as a relatively autonomous production unit, with the overarching objective of maximizing welfare. As an entity distinct from both the corporate and public sectors, the household comprises specific family members, possesses its own livelihood assets, and employs distinct livelihood strategies. The fundamental objective of household livelihood activities is to maximize welfare, encompassing the augmentation of household income, enhancement of consumption levels, and the overall improvement of welfare standards.

This characteristic determines that in livelihood research, the family is the core actor. Secondly, as the main body of livelihood activities, families carry out livelihood activities in specific ecological and institutional environments and are influenced by external shocks. Both ecological and institutional environments can have an impact on family livelihoods. From the perspective of the ecological environment, seasonal ecological changes, ecological fragility, and environmental carrying capacity determine the difficulty of family livelihoods [12, 13]. It is relatively difficult to carry out livelihood activities in areas with severe ecological environmental changes and obvious ecological fragility, such as high-altitude mountains and desertification, and the overall level of well-being is also relatively low [14]. On the contrary, carrying out livelihood activities in areas with good ecological environments such as plains will result in a higher overall level of well-being. In addition, public services, as a unique livelihood asset, are mainly influenced by institutional decision-making processes.

## Research design

### Methodology

Utilizing a large-scale survey database and quantitative models, this paper aims to explore the relationship between health coverage medical system and citizens health. Compared with cross-sectional data regression analysis, data regression analysis has the following three outstanding advantages: first, higher coefficient estimation accuracy. Compared with cross-sectional data, data increases the information of individual dimension and trial dimension, and the sample size is larger, which can provide higher progress and accuracy for coefficient estimation and robustness test. Second, reduce the problem of missing variables to a certain extent. Missing variable bias has always been a prominent problem in cross-sectional data regression model. Third, data provides more dynamic information for respondents, which is conducive to causal inference. In terms of the amount of information, the cross-sectional data can not provide the information of the respondents in the time dimension, and many research topics need to be analyzed in the time dimension.

For instance, in examining the impact of healthcare coverage on the multidimensional poverty of families, it is noteworthy that the policy effect of such coverage often necessitates a prolonged manifestation, thereby posing challenges for in-depth cross-sectional research. Furthermore, data holds significant advantages in causal inference, where chronological order serves as a fundamental prerequisite. The fundamental principle of causality posits that the cause precedes the effect. Consequently, the absence of temporal dimensions in data, which would enable the discernment of pre- and post-conditions, might lead to spurious causalities in coefficient estimation and statistical inference. In reality, the temporal information contained in data offers valuable support for investigating topics such as population migration, individual educational trajectories, job mobility, family dynamics, sustainable household livelihoods, social stratification, and the evaluation of public policy effects, thereby exhibiting rich potential for application.

Based on the theoretical framework of sustainable livelihoods and the classic health demand model [15], this paper discusses the impact of healthcare coverage on health. From the perspective of initial health stock, Grossman proposed a way to analyze the change of health asset[15]:

$$H_{i + 1} \, - \,H_{i} \, = \,I_{i} \, - \,\delta_{i} H_{i}$$

${H}_{i+1}$ is the current health status and is also the dependent variable of this paper. Current health status, subject to the initial health stock ${H}_{i}$. ${\delta }_{i}$ is the health depreciation rate in period I, and its value range is 0 to 1. Factors such as age, education level, living environment and living habits will affect the health depreciation rate. Most of the relevant studies have analyzed the changes of health asset from the perspectives of respondents' gender, age, education level, living environment health, smoking and alcohol abuse ${\delta }_{i}{H}_{i}$ discuss. Health depreciation rate affects the initial health asset stock ${H}_{i}$. Ultimately affect the current health status. In addition, health investment ${I}_{i}$ is also an important factor affecting the current health status. The specific types of health investment are healthcare coverage investment, health food investment, drug investment, nutrition investment and investment in improving the living and sanitation environment. In fact, healthcare coverage is a typical health investment. Although Grossman did not consider healthcare coverage as a health investment in his research, healthcare coverage participation is essentially a health investment. On the one hand, healthcare coverage investment will occupy the current consumption funds. Healthcare coverage can only play a role when medical expenses occur, so it can be regarded as an investment behavior. On the other hand, the positive effect of healthcare coverage on health status has received more attention.

### Data and variables

The Chinese national longitudinal study on household sustenance is orchestrated and executed by the China Social Sciences Research Center. This study involves a biennial assessment of both household and adult demographics. Commencing in 2010, the foundational survey serves as the reference for subsequent data collection. The initial survey's dataset encompasses a diverse array of regions, spanning 25 provinces, municipalities, and autonomous regions. It includes data from 635 villages, representing 14,960 households, 33,600 adults, and 8,990 children. It is noteworthy that, in alignment with ethical research standards and to uphold privacy, the database disseminates only the provincial geographic codes for the entire spectrum of family and adult samples. The identifiers for the more specific levels of prefecture-level cities, districts, and counties have been obscured through randomization. Consequently, researchers are precluded from correlating the data with other specific regional information at the prefecture, district, or county levels.

In terms of sampling methods, the China Household Tracking Survey employs a stratified random sampling technique, which bolsters the national representativeness of the sample （Table 1）. The 2010 baseline survey for this longitudinal study encompasses a selection of 25 provinces, whose collective population constitutes 94.5% of the total populace of mainland China, thereby rendering the database a microcosm of the nation's demographic. The survey delineates five "major provinces" with an intended sample size of 1,600 households each, and the remaining 20 "minor provinces" aim for a sample size of 8,000 households apiece, ensuring a comprehensive and balanced reflection of the country's household dynamics.

Furthermore, the implicit stratification multi-stage probability sampling methodology ensures the representative nature of the family sample. Specifically, the household sampling process unfolds in three distinct stages. Firstly, the selection of survey provinces occurs, wherein provincial units are differentiated into 'large provinces' and 'small provinces' through official administrative division data, utilizing the principle of implicit stratification. Shanghai, due to its unique status, is categorized as a 'large province'. In the second stage, the selection of survey cities is undertaken, with provincial capital cities serving as the initial implicit stratification. Other prefecture-level cities are then evaluated based on socioeconomic status (SES), local per capita GDP, population density, and the proportion of non-agricultural population. Lastly, in the third stage, residents within the selected villages and communities are surveyed. Sampling of households within these communities and villages is conducted using a household list sampling frame generated by a geographic information system, along with the random starting point equidistant circular sampling technique. From each village or community, 25 households are selected for inclusion in the study.

The database boasts an expansive sample size, with a dynamic integration of longitudinal and annually introduced samples. To ensure a substantial pool of data in the Chinese Household Tracking Survey database over an extended period, the research team strategically replenishes the sample annually to counteract attrition. This approach ensures that each data collection phase includes both the original baseline survey samples and newly integrated samples. The dual strategy serves two key purposes: firstly, it facilitates stable tracking of baseline participants, yielding a wealth of comprehensive data for longitudinal family dynamics studies in China. Secondly, it acknowledges the international research consensus that sample attrition is an inevitable aspect of follow-up surveys. To maintain the database's capacity to offer nuanced family sample data over time, the survey annually incorporates new samples and continues tracking in subsequent years. Consequently, the foundational database of China's household tracking survey is essentially a hybrid of cross-sectional and longitudinal samples. For the sake of data management reliability, the project team disseminates data on an annual basis. It is evident that extracting a pure balanced panel from the raw data of the publicly available mixed samples necessitates extensive data processing efforts. In terms of database categorization, there are four principal types: the adult database, which captures individual adult data; the family database, encompassing household-level information; the village database, focusing on community-level data; and the children's answer database, which compiles responses from younger participants. Each type is meticulously curated to support a wide array of academic and policy-oriented research inquiries.

The dependent variable under investigation is the state of public health. The degree of health is a pivotal metric for gauging the extent of capability poverty and stands as a key measure of the general health of a population. It is a fundamental prerequisite for any societal member to engage in sustenance activities and to generate income, necessitating good health. The level of health is thus a critical component in assessing the overall health of a populace. Consequently, this study adopts health level as a significant proxy for evaluating population health. In terms of the measurement techniques employed, this research primarily relies on self-assessed health as the metric. Self-rated health is a crucial approach for quantifying the health level. The subjective evaluations provided by respondents offer insights into dimensions of health that may not be captured by purely objective criteria. This method is valuable as it taps into the individual's perception of their own health status, which can be indicative of aspects that are not readily quantifiable through empirical measures alone.

The independent variable in question is the extent of healthcare coverage. This study concentrates on two principal metrics for assessing healthcare coverage: the rate of insurance enrollment and its resultant influence on personal conduct. In the context of China's New Rural Cooperative Medical Scheme, it has been observed that those enrolled in the insurance system are markedly more inclined to seek medical care at hospitals when confronted with health issues or potential illness, as opposed to those who are uninsured. The provision of healthcare coverage mitigates financial constraints by subsidizing a segment of medical expenses, which in turn lessens the pressure on household budgets and promotes the development of sustainable livelihoods over time. Furthermore, the advancement of China's medical insurance system has enhanced the confidence of economically disadvantaged households in participating in income-generating activities, such as making small-scale investments and undertaking business expansions. The presence of this system provides a psychological safety net for impoverished families, enabling them to secure reimbursement for medical expenditures through their insurance coverage. This research has noted that in areas with robust participation in the New Rural Cooperative Medical Scheme, there is an increased tendency among impoverished households to diversify their livelihood investments. Importantly, a cornerstone of the "health poverty alleviation" strategy is to ensure comprehensive healthcare coverage for impoverished families through targeted investments. The central government has earmarked specific financial transfers to regions characterized by poverty, thereby aiding in the provision of fundamental medical services to these households. This approach underscores the significance of healthcare coverage as a catalyst for poverty reduction and a means to empower vulnerable populations to pursue economic self-sufficiency and well-being.

The explanatory variable under examination is categorized. The initial dataset of the Chinese family tracking survey included five distinct categories of healthcare coverage: public medical care, urban employee insurance, urban resident insurance, supplementary healthcare coverage, and the new rural cooperative medical system. However, within the current medical security framework in China, three types of coverage are predominantly recognized: urban employee insurance, urban resident insurance, and the new rural cooperative medical system. Although supplementary healthcare coverage and public medical care are significant components of the broader medical coverage, they are not the central elements of China's fundamental medical security system. Considering the relatively lower overall coverage rates for public medical care and supplementary insurance in China, coupled with the limited sample size within the dataset, this study narrows its focus to the three principal types of healthcare coverage: urban employee insurance, urban resident insurance, and the new rural cooperative medical system. This approach allows for a more in-depth analysis of the most prevalent forms of medical coverage and their impact on the population's health and economic well-being. By concentrating on these three categories, the study aims to provide a clearer understanding of how different healthcare coverage options influence individual health behaviors and outcomes within the context of China's evolving medical security landscape.

The confounding variables in this study are categorized into individual-level and family-level variables. At the individual level, the primary control variables encompass gender, age, education level, and smoking habits. Gender, age, and education are demographic variables that are commonly controlled for in standard models. Smoking is represented as a binary variable, and it is recognized as a significant determinant when examining factors that influence an individual's health status, as posited by Grossman (1972). Shifting focus to the family level, the control variables are primarily derived from the livelihood asset pentagon within the theoretical framework of sustainable livelihoods. This framework comprises five distinct assets: ecological, material, marketable, human, and community resources. The ecological asset is measured by the market value of household land assets. The material asset is assessed by the type of residential housing. The marketable asset is quantified by the market value of household assets that can be sold or traded. The human asset is gauged by the average years of schooling among family members. Lastly, community resources are operationalized using a binary variable.

By incorporating these multifaceted variables, the study seeks to account for a comprehensive set of factors that may confound the relationship between healthcare coverage and population health. This approach allows for a nuanced understanding of how individual behaviors and family resources intersect to influence health outcomes, thereby providing a robust basis for analysis and policy implications (Table 1).

**Table 1:** The sample distribution of family livelihood survey

Sample unit	Provinces, municipalities and autonomous regions
Big provinces	Shanghai, Liaoning, Henan, Gansu, Guangdong
Small provinces	Hunan, Sichuan, Guizhou, Yunnan, Tianjin, Beijing, Chongqing, Shaanxi Jiangsu, Zhejiang, Anhui, Shandong, Hebei, Shanxi, Jilin, Hubei, Fujian, Jiangxi,

Source: China's household survey technical report series-1

## Empirical analysis

### Descriptive analysis

For an overview of the livelihood assets and strategies as depicted in the data from the China Household Tracking Survey, please refer to Table 2. This table presents a descriptive statistical analysis that encapsulates the various components of livelihood assets and the strategic approaches adopted by households in managing their resources and opportunities. It offers a snapshot of the economic and social dynamics at the household level, providing insights into the distribution and utilization of ecological, material, marketable, human, and community resources across the surveyed population.

**Table 2:** Descriptive statistical analysis of livelihood asset and livelihood strategies

Livelihood asset	Mean	Standard deviation	Min	Max
Material asset	2.80	1.86	1.00	6.00
Ecological asset	23651.48	82519.71	0.00	5015625.00
Marketable asset	38892.91	142168.60	0.00	5000000.00
Community resource	0.51	0.50	0.00	1.00
Human resource	7.28	5.15	0.40	56.00
Livelihood strategy	0.45	0.50	0.00	1.00

In the realm of ecological assets, the mean value stands at 23,651 yuan, with the lowest recorded value being 1 yuan and the highest peaking at 5,015,625 yuan. These figures represent the market value of land assets owned by households, and the data is categorized at the ratio level, indicating a fixed unit of measurement. For marketable assets, the average value is calculated to be 38,892.91 yuan, with the minimum value at 0 yuan and the upper limit reaching 5 million yuan. This category also pertains to assets that can be readily traded or sold in the market, and like ecological assets, these are measured using a fixed ratio scale in terms of market value. Community resources are measured using a binary variable, with both the minimum and maximum values being 0.51. This dummy variable serves to indicate the presence or absence of community resources, with 1 signifying the availability and 0 indicating the lack thereof. The human asset is quantified by the average years of schooling within the family, which is 7.28 years. This figure ranges from a minimum of 0.4 years to a maximum of 56 years. As a ratio-level variable, it reflects the educational attainment within the household, which is a critical component of human capital. Material assets are represented by an ordered variable with an average value of 2.8. The scale for this variable ranges from 1 to 6, indicating different levels or types of residential housing owned by the households. Lastly, the overall estimate of diversified livelihood strategies is 0.45, suggesting that approximately 45% of the surveyed families have embraced a diversified approach to their livelihood strategies. This variable is also a binary indicator, with values of 0 and 1 representing the absence and presence of such strategies, respectively. These descriptive statistics provide a comprehensive overview of the various livelihood assets and strategies among the households in the China Household Tracking Survey, offering valuable insights into the economic and social dynamics that influence household well-being and resilience (Fig. 1).

From an individual household standpoint, livelihood outcomes are predominantly manifested in subjective well-being, self-rated health, total medical expenditures, and out-of-pocket medical costs. Subjective well-being is categorized as an ordinal variable, with a scale ranging from 1 to 5. The mean score of 3.74 suggests that respondents generally report a high level of life satisfaction. Self-rated health, another important indicator, is measured on a fixed interval scale, with possible values also ranging from 1 to 5. The average score of 2.82 among respondents in the China Household Tracking Survey indicates that there is considerable scope for enhancing perceived health levels. This suggests that while life satisfaction may be relatively high, the self-assessed health status points to areas that require attention and potential interventions to improve overall public health. These variables are critical for understanding the multidimensional aspects of livelihood outcomes, as they encompass both emotional and physical well-being, as well as the financial implications of healthcare. The contrast between the relatively high life satisfaction scores and the lower self-rated health scores underscores the complexity of factors influencing livelihood outcomes and highlights the need for a nuanced approach to policy and intervention strategies aimed at improving health and well-being.

## Regression model analysis

In Equation I, a spectrum of individual-level covariates was integrated, including chronological age, gender, educational qualifications, and tobacco usage. The quantitative analysis disclosed that, upon consideration of personal characteristics and the invariant attributes of temporal and provincial influences, the involvement in medical insurance schemes had a pronounced effect on health outcomes. To be specific, the coefficient for medical insurance participation was estimated at 0.305, with a stringent standard error of 0.018, signifying a statistically significant relationship at the 99.9% confidence threshold. This suggests that individuals with medical insurance tend to have a more favorable health status relative to those uninsured. When examining the individual-level covariates, the coefficient for age was estimated at 0.024, with a p-value significantly less than 0.000, indicating a positive link between age and health. Conversely, the coefficient for gender was -0.201, also significant at the 99.9% confidence threshold, which implies that male individuals are more likely to report enhanced health levels than their female counterparts. Moreover, the coefficient for educational level was 0.011, significant at the 99.9% confidence level, denoting a positive relationship between the level of education and self-assessed health. Additionally, the coefficient for smoking behavior was -0.136, with a p-value well below 0.000, underscoring the adverse effect of smoking on health status. In terms of the model's predictive efficacy, the R^2^ value for Equation I was recorded at 0.122. The intra-class R^2^ was 0.113, suggesting a moderate explanatory capacity within individual groups. The inter-class R^2^ was slightly elevated at 0.144, indicating a somewhat stronger explanatory capacity when comparing between groups. Collectively, these R^2^ values indicate that while the model possesses a significant, yet not flawless, capability to predict health outcomes, there is room for further refinement to enhance its explanatory and predictive strengths.

Equation II expands the analysis to include household-level control variables, which are associated with the metrics of livelihood assets. The statistical synthesis indicates that healthcare coverage continues to exert a significant influence on health outcomes, even when variables related to livelihood assets are controlled for. Specifically, the coefficient for healthcare coverage participation is estimated to be 0.264, with a standard error of 0.018, which is statistically significant at the 99.9% confidence level. This finding underscores the substantial health benefits conferred by healthcare coverage. In the context of the livelihood asset control variables, the material asset shows a statistically significant positive effect on self-rated health, with an estimated coefficient of 0.020, also significant at the 99.9% confidence level. The ecological asset, however, does not have a discernible impact on self-rated health. The marketable asset, on the other hand, has a significant positive effect, with a coefficient of 0.002 that is significant at the 99.9% confidence level, indicating that assets that can be readily converted to cash positively correlate with health status. Additionally, the community resource variable, with an estimated coefficient of 0.079 and a standard error of 0.026, achieves statistical significance at the 99% confidence level. This suggests that access to community resources positively affects health status. Similarly, the human asset, measured by the average years of education within the household, has an estimated coefficient of 0.186 and a standard error of 0.017, significant at the 99.9% confidence level, highlighting the pivotal role of education in improving health outcomes. It is also noteworthy that the adoption of diversified livelihood strategies significantly and positively impacts health status, with the estimated coefficient being significant at the 99.9% confidence level. This points to the advantages of having a variety of livelihood approaches for enhancing overall well-being.

Equation III integrates a comprehensive array of control variables at both the individual and family levels. Despite potential constraints in the model's estimation and the necessity to consider a broad spectrum of relevant variables, healthcare coverage emerges as a significant and positive predictor of health status. The coefficient linked to healthcare coverage participation is estimated at 0.292, which is statistically significant at the 99.9% confidence level. This result highlights the enhanced health conditions among individuals with healthcare coverage relative to the uninsured, reaffirming the considerable health advantages of such coverage. In the realm of individual-level control variables, the coefficient for age is found to be 0.025, which is statistically significant at the 99.9% confidence level. This positive association between age and health status may be indicative of the cumulative effects of experience and resource accumulation over an individual's lifetime. In contrast, the coefficient for gender is estimated at −0.025, also reaching statistical significance at the 99.9% confidence level. This suggests that, on average, male individuals report better health conditions than their female counterparts, which could reflect underlying gender disparities in health outcomes that merit further investigation and policy attention. The inclusion of both individual and family-level variables in Equation III allows for a more nuanced understanding of the multifaceted determinants of health. By accounting for a wide range of factors that could influence health status, the model provides a robust framework for analyzing the impact of healthcare coverage and other covariates on individual health outcomes. This comprehensive approach is essential for developing well-informed policies and interventions aimed at improving public health and addressing health inequalities. The estimated coefficient pertaining to education level stands at 0.015, achieving significance at a 90% confidence level. This finding underscores a positive association between educational attainment and self-rated health, suggesting that individuals with higher educational levels tend to report superior health status. Turning to household-level control variables, material asset exhibits a notable positive influence on self-rated health, with an estimated coefficient of 0.019, significant at a 99% confidence level. Additionally, the coefficient for the impact of marketable asset on self-rated health is 0.005, achieving significance at the 99.9% confidence level. This indicates that marketable assets have a substantial positive effect on self-rated health. Furthermore, the estimated coefficient for community resource is 0.201, attaining significance at the 99.9% confidence level. This highlights the significant positive impact of community resources on health status. Lastly, the estimated coefficient for human asset is 0.015, with a standard error of 0.003. The associated impact coefficient is significant at a 99.9% confidence level, indicating that human assets also exert a notable positive influence on health status.

Regarding the adoption of livelihood strategies, the coefficient is calculated to be 0.344, coupled with a standard error of 0.015. This coefficient holds statistical significance at the 99.9% confidence level, denoting that the implementation of diversified livelihood strategies is positively and significantly associated with improved health status. This finding suggests that households employing a variety of approaches to sustain their livelihoods are more likely to experience better health outcomes, underscoring the importance of economic diversification as a contributor to overall well-being.

## Conclusion and discussion

One of the pivotal roles of healthcare coverage is to offer health security for its enrollees. This study delves into the health implications of healthcare coverage through various lenses, employing fixed effects modeling grounded in the sustainable livelihoods framework, and leverages extensive datasets from Chinese household longitudinal surveys. The investigation employs a multifaceted approach to understand how healthcare coverage influences health outcomes, considering both individual and household-level factors. By applying fixed effects models, the study controls for unobserved heterogeneity that could bias the results, thus providing a more accurate estimation of the relationship between healthcare coverage and health status. The sustainable livelihoods theory provides a comprehensive framework for this analysis, emphasizing the importance of various capitals—natural, physical, financial, human, and social—in determining the livelihood outcomes of individuals and households. This theory guides the examination of how healthcare coverage, as a form of financial and social capital, contributes to the health and well-being of the population. The use of large-scale data from Chinese household tracking surveys allows for robust statistical analysis and generalizable findings. These surveys offer rich and detailed information on individuals and households, including their healthcare coverage, health status, and livelihood strategies, which are crucial for understanding the complex dynamics between healthcare coverage and health outcomes. Overall, this study contributes to the literature by providing a nuanced understanding of the health effects of healthcare coverage in the context of China, highlighting the significance of considering sustainable livelihoods in health policy and program design. The findings can inform strategies to enhance healthcare coverage and improve public health outcomes.

This paper reveals that individuals enrolled in basic healthcare coverage experience substantial health benefits compared to those who remain uninsured. The salutary impact of healthcare coverage is examined and confirmed through three distinct dimensions: first, the distinction between the insured and the uninsured. The research establishes that healthcare coverage, irrespective of its specific form, confers a measure of health benefits to those who are insured. Second, variations in the types of insurance participation. The study discerns that, when considering marginal effects, healthcare coverage designed for urban employees exerts the most substantial positive influence on health status. This is followed by coverage for urban residents, whereas the new rural cooperative medical system appears to have a comparatively lesser impact on health outcomes. Third, the heterogeneity in the health effects of healthcare coverage. The research uncovers significant disparities, particularly highlighting the pronounced health benefits of healthcare coverage among the elderly population aged 60 and above. This suggests that healthcare coverage is particularly effective in providing health protection for this demographic. Additionally, an urban–rural divide is observed, with urban residents experiencing greater health benefits from healthcare coverage participation than their rural counterparts. This is reflective of the types of healthcare coverage typically accessed by these populations, with urban residents predominantly enrolled in urban-based healthcare schemes and rural residents mainly participating in the new rural cooperative healthcare coverage. It is noteworthy that despite China's gradual initiation and expansion of the integration of urban resident healthcare coverage since 2016, the process of provincial-level coordination has been sluggish, as indicated by data from Chinese household tracking surveys. This underscores the need for continued efforts to streamline and integrate healthcare systems to ensure equitable access and benefits across different population segments and geographical areas.

This article builds upon the existing body of knowledge by offering a nuanced perspective on the subject. While previous studies have documented the health benefits linked to healthcare coverage, this research introduces an additional layer by emphasizing its role in poverty alleviation related to health. It highlights how healthcare coverage schemes contribute to positive health outcomes by providing a financial safety net for individuals, thereby encouraging them to seek necessary medical care without the fear of incurring prohibitive costs. The reimbursement mechanism within these coverage schemes serves as an incentive for insured individuals to access healthcare services promptly. This is particularly crucial in preventing the escalation of minor health issues into more severe conditions, a common occurrence in rural China due to economic constraints. The study's examination of China's new rural cooperative medical system illustrates that insured farmers are considerably more proactive in seeking medical attention for ailments, reducing the risk of complications arising from untreated illnesses. Moreover, healthcare coverage plays a pivotal role in economic stability by subsidizing medical expenses, which in turn lessens the financial burden on households. This support is instrumental in nurturing sustainable livelihood development over time. As the medical coverage system in China has evolved, it has fortified the confidence of economically disadvantaged families, empowering them to engage in small-scale investments and enhance their productive capacities. The integration of healthcare coverage into the social fabric has thus emerged as a multifaceted tool, not only improving health outcomes but also acting as a catalyst for economic empowerment and poverty reduction. This research contributes to the discourse by elucidating the intricate relationship between healthcare coverage, health equity, and economic development, offering valuable insights for policymakers and stakeholders in the healthcare sector.

## Data Availability

The datasets used and/or analyzed during the current study are available from the corresponding author upon reasonable request.
